# Combined Self-Nanoemulsifying and Solid Dispersion Systems Showed Enhanced Cinnarizine Release in Hypochlorhydria/Achlorhydria Dissolution Model

**DOI:** 10.3390/pharmaceutics13050627

**Published:** 2021-04-28

**Authors:** Ahmad A. Shahba, Ahmad Y. Tashish, Fars K. Alanazi, Mohsin Kazi

**Affiliations:** 1 Kayyali Chair for Pharmaceutical Industries, College of Pharmacy, King Saud University, Riyadh 11451, Saudi Arabia; shahba@ksu.edu.sa (A.A.S.); afars@ksu.edu.sa (F.K.A.); 2 Department of Pharmaceutics, College of Pharmacy, King Saud University, Riyadh 11451, Saudi Arabia; wtashish@yahoo.com

**Keywords:** self-nanoemulsifying drug delivery systems (SNEDDS), solid dispersion, poorly-water soluble drugs, hypochlorhydria/achlorhydria, solidification, elevated gastric pH

## Abstract

The study aims to design a novel combination of drug-free solid self-nanoemulsifying drug delivery systems (S-SNEDDS) + solid dispersion (SD) to enhance cinnarizine (CN) dissolution at high pH environment caused by hypochlorhydria/achlorhydria. Drug-loaded and drug-free liquid SNEDDS were solidified using Neusilin^®^ US2 at 1:1 and 1:2 ratios. Various CN-SDs were prepared using freeze drying and microwave technologies. The developed SDs were characterized by differential scanning calorimetry (DSC) and X-ray powder diffraction (XRD). In-vitro dissolution studies were conducted to evaluate CN formulations at pH 6.8. Drug-free S-SNEDDSs showed acceptable self-emulsification and powder flow properties. DSC and XRD showed that CN was successfully amorphized into SDs. The combination of drug-free S-SNEDDS + pure CN showed negligible drug dissolution due to poor CN migration into the formed nanoemulsion droplets. CN-SDs and drug-loaded S-SNEDDS showed only 4% and 23% dissolution efficiency (DE) while (drug-free S-SNEDDS + FD-SD) combination showed 880% and 160% enhancement of total drug release compared to uncombined SD and drug-loaded S-SNEDDS, respectively. (Drug-free S-SNEDDS + SD) combination offer a potential approach to overcome the negative impact of hypochlorhydria/achlorhydria on drug absorption by enhancing dissolution at elevated pH environments. In addition, the systems minimize the adverse effect of adsorbent on drug release.

## 1. Introduction

Hypochlorhydria, a major public health problem which is characterized by diminished or even absent (achlorhydria) gastric acid secretion [1]. The most common cause of spontaneous hypochlorhydria is chronic atrophic gastritis, which can be caused by either H. pylori infection or an autoimmune reaction. More than 30% of individuals aged over 60 years are affected by chronic gastritis which implies that hypochlorhydria is one of the most common pathologies in the aged population. In addition, prolonged use of proton pump inhibitors substantially increases the prevalence of hypochlorhydria, especially in the elder population. Furthermore, hypochlorhydria is common in patients with gallbladder disease and particularly patients with gallstones [2]. Bariatric surgery significantly reduces gastric capacity leading to significant drop in gastric acid secretions and therefore causing elevated pH level within the remaining gastric pouch [3,4].

All the aforementioned conditions lead to significant elevation of gastric pH level and could potentially affect oral bioavailability of weakly basic poorly-water soluble drugs (WB-PWSDs), particularly those, which possess pH-dependent solubility [5,6]. At normal gastric conditions, the WB-PWSD experiences its first exposure at the lower pH conditions in the stomach which favor good drug solubilization. Subsequently, upon shifting to the intestinal environments, drug tends to precipitate due to unfavorable higher pH conditions. However, in the body, this phenomenon might be diminished due to sink conditions and fast absorption capacity available in the intestinal tract.

At hypochlorhydria/achlorhydria conditions, the WB-PWSDs are initially expected to approach the unfavorable high pH environment from the gastro-intestinal tract. Therefore, this drug would be deprived from its favorable low pH environments, leading to extremely poor drug solubility and dissolution at higher pH conditions. Such drug properties would lead to exponential decline in drug solubility, dissolution and bioavailability upon gastric pH increase. Several weakly basic drugs such as atazanavir, Itraconazole and ketoconazole experienced significant reduction in drug absorption and systemic exposure upon gastric pH elevation [6].

Liquid self-nanoemulsifying drug delivery systems (SNEDDS) is very valuable option for formulating WB-PWSD because it provides substantial enhancement of drug dissolution and bioavailability with less likely pH-induced effects on dissolution. However, liquid SNEDDS suffers from several limitations such as capsule shell incompatibilities, formulation leakage risk, oil oxidation and possibility of drug precipitation. Furthermore, some drugs may chemically degrade in presence of SNEDDS excipients. In contrast, Solid SNEDDS are able to circumvent liquid SNEDDS limitations, maintain the SNEDDS-induced enhanced drug solubilization along with greater stability, improved patient compliance of the solid dosage forms [7,8]. Adsorption onto high surface area inorganic silica materials has been commonly used to solidify liquid SNEDDS into free-flowing powders. However, this technique has been associated with significant retardation of drug release from the formulation. Several studies have presented the negative impact of adsorbent on drug release from solidified SNEDDS [9,10]. Compared to >80% drug release from liquid SNEDDS, some studies showed that the adsorbents significantly reduced drug release to as low as 20% [11].

More than one mechanism could be involved in the phenomenon of the loss in drug release extent upon SNEDDS adsorption onto inorganic silica carriers. The decreased drug release could be due to gel formation which clogs the meso pores of the silicate, thus trapping the liquid SNEDDS inside [12]. Another explanation might be based on SNEDDS retention within the mesoporous regions (pore size = 2–50 nm), which do not have enough room for emulsification compared to the macro porous structure of the adsorbent (pore size > 50 nm) which provide more physical space for emulsification process. Some studies also suggested the development of physical bonds between the drug and carrier might favor the diffusion of the drug from the SNEDDS to the surface of the adsorbent followed by nucleation and drug precipitation which in turn retards complete drug release from the solid SNEDDS [13,14].

**Figure 1:**
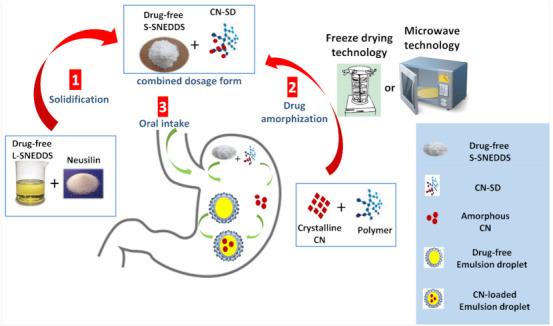
Schematic diagram of the manufacturing and performance of (CN-SD + drug free S-SNEDDS) combined dosage form. CN-SD denotes: cinnarizine-loaded drug dispersion; S-SNEDDS: solid self-nanoemulsifying drug delivery systems.

Accordingly, new approaches are highly demanded to enhance drug release from solidified SNEDDS. Therefore, the current study introduces a new technique for SNEDDS solidification using combination of drug-free S-SNEDDS with drug-loaded solid dispersion, prepared by microwave irradiation or freeze drying technique (Figure 1). Such innovative technique is still new and, to our best knowledge, it has not been reported yet in any publications. This system is based on drug separation from S-SNEDDS during manufacturing and storage. On the other hand, upon oral administration and formulation exposure to aqueous media (Figure 1), the following two main steps were hypothesized to take place as follows:

(1) self-emulsification of the drug-free S-SNEDDS leading to the formation of drug-free nanoemulsion;
(2) simultaneously partitioning of the amorphized drug inside the formed nanoemulsion droplets, leading to enhanced drug dissolution: this system offers the advantage of avoiding the unfavorable drug interaction with the adsorbent (during storage) that could retard complete drug release from S-SNEDDS [13,14].

Cinnarizine (CN), a WB-PWSD, suffers from two major limitations—pH-dependent poor aqueous solubility and chemical instability in SNEDDS excipients—even after solidification [15,16]. Therefore, it is expected to show very poor dissolution within hypochlorhydria/achlorhydria conditions due to the drug exposure to higher pH environments at its initial approach to the gastrointestinal tract [17]. The purpose of the current work is to develop a combined system of drug-free S-SNEDDS and CN-SD and evaluate its role in enhancing drug release compared to the conventional CN loaded S-SNEDDS. Within the scope of the current study, CN formulations were challenged at high pH dissolution environment (pH 6.8) due to hypochlorhydria/achlorhydria conditions [3,4]. Furthermore, the two novel techniques, namely, freeze drying and microwave-induced (SD + drug-free solid SNEDDS), were investigated for their potential role to enhance CN release from solidified SNEDDS.

## 2. Materials and Methods

### 2.1. Materials

Imwitor 308 (I308, Glyceryl Caprylate, monoglycerides ≥ 80%) was kindly supplied by Sasol Germany GmbH (Werk Witten, Germany). Oleic acid (OL, long chain fatty acid C18:1) was obtained from Avonchem (Cheshire, UK). Cremophor EL (Cr-El, polyoxyl 35 castor oil) was supplied by BASF (Ludwigshafen, Germany). Cinnarizine (CN, purity > 99.5) was supplied by FDC Limited (Maharashtra, India). Fish gelatin capsules (size 0) were donated by Capsugel (Morristown, NJ, USA). Vivapharm^®^ Hydroxy-propyl methyl cellulose (HPMC) E3, was purchased from JRS Pharma (Rosenberg, Germany). Kollidon K30^®^ (Polyvinylpyrrolidone, PVP) was donated by BASF (Ludwigshafen, Germany). Pluronic F127 (PL-127, polyoxyethylene-polyoxypropylene block co-polymer) was purchased from Sigma Aldrich, St. Louis, MO, USA. Neusilin^®^ US2 (amorphous magnesium aluminometasilicate) was obtained from Grace (Worms, Germany).

### 2.2. Preparation of Drug-Free and Drug-Loaded Liquid Self-Nanoemulsifying Drug Delivery Systems (L-SNEDDS)

Based on recent publications [18], drug-free L-SNEDDS was prepared using OL/I308/Cr-El (at 25/25/50, *w*/*w* ratios) (Table 1). Initially, the co-surfactant (I308) was preheated at 40 °C, for ≈30 min, to ensure complete melting and homogenization. Then, the melted co-surfactant was added to the remaining excipients. Finally, the components were thoroughly mixed using L32 Labinco magnetic stirrer (Labinco BV, Breda, The Netherlands) at ≈1250 rpm for ≈10 min [19,20].

In drug-loaded L-SNEDDS, CN was dissolved in the prepared formulations at 80 mg/g concentration (Table 1), which represents ≈90% of the equilibrium solubility of CN in SNEDDS (88 mg/g) [19]. Then, the mixture was thoroughly mixed as described earlier.

### 2.3. Preparation of Drug-Free and Drug-Loaded Solid Self-Nanoemulsifying Drug Delivery Systems (S-SNEDDS)

A predetermined amount of the adsorbent Neusilin^®^ US2 was added to L-SNEDDS (1 g) at 1:1 and 2:1 *w*/*w* ratios for S-SNEDDS (1× NUS) and (2× NUS), respectively (Table 1). Then, the mixture was thoroughly mixed using L32 Labinco magnetic stirrer (Labinco BV, Breda, The Netherlands) at ≈700 rpm for ≈10 min, until a uniform solid powder was obtained [21]. Subsequently, the solidified SNEDDS were characterized to achieve the optimum formulation, as discussed below.

### 2.4. Preparation of Drug Loaded Solid Dispersions (SD)

#### 2.4.1. Freeze Dried Solid Dispersion (FD-SD)

Binary systems of CN and HPMC E3 at 1:4 *w*/*w* ratio were co-dissolved in 0.12 M HCl solution at 3% *w*/*v* total solid concentration. The solution was efficiently stirred until a clear solution was obtained to ensure complete CN solubilization. The resultant solutions were freeze-dried at −60 °C (Alpha 1-4 LD Plus, Osterode am Harz, Germany) for at least 48 h [22,23]. The resulting solidified residue was grinded by mortar and pestle and passed through 315 µm sieve to obtain a uniform size fine powder.

#### 2.4.2. Microwave Irradiation Solid Dispersion (MW-SD)

MW-SD were prepared using domestic MW irradiation (Samsung Model ME0113M1) [24]. Pure drug was blended with PL-127 at 1:9 *w*/*w* ratio (Table 1). A precise amount of the drug and the carrier (approximately 1 g) were gently mixed for about 1 min. The MW power was set at power 900 W and the samples were subjected to MW irradiation one by one at the exact place. Upon melting, the samples were allowed to be cooled and solidified then placed in the desiccator for at least 24 h to remove any residual moisture. Later, samples were pulverized and sieved to obtain uniform powder and stored in air-tight tubes for further use [24,25].

#### 2.4.3. Preparation of Combined Formulation (SD + S-SNEDDS)

Irrespective of the solid dispersion technique, SD (equivalent to 25 mg CN) was combined with drug-free solid SNEDDS to maintain CN at ≈80% of its equilibrium solubility in SNEDDS.

#### 2.4.4. Determination of CN Encapsulation Efficiency

A predetermined amount of CN-SD (equivalent to ≈2 mg CN) was weighed and transferred into a 25 mL volumetric flask. Then, the flask was filled with 1% HCL up to the mark. The mixture was sonicated for 45 min to ensure complete drug solubilization. Further, an aliquot of the mixture was transferred into 1.5 mL Eppendorf tube and centrifuged using Benchtop centrifuge (PrO-Research K2015, Centurion Scientific Ltd., Chichester, UK) at 10,000 rpm for 5 min. Supernatant (0.25 mL) was diluted in acetonitrile and assayed by a validated UPLC assay [26]. A minimum of three replicates was considered for each sample.

### 2.5. Optimization and Characterization of the Combined Formulation

#### 2.5.1. Powder Properties

Bulk and tapped densities of solid SNEDDS were measured using tapped density tester-USP (Erwika SVM 102); 10 g of the sample was poured into a 100 mL graduated cylinder with 0.5 mL markings. Furthermore, the compressibility index and Hausner ratio were calculated [27,28,29]. In addition, the angle of repose was determined using the height funnel method [28,29].

#### 2.5.2. Differential Scanning Calorimetry (DSC)

CN-SDs were examined using Differential scanning calorimeter equipped with auto sampler and chiller (DSC8000, Elmer, Waltham, MA, USA). Accurately weighed samples (3–5 mg) were placed in aluminum pans and hermetically sealed using crimp sealer. The sealed sample pans were heated against blank aluminum pan from 25 to 200 °C, at 10 °C/min heating rate and under 50 mL/min nitrogen gas flow rate. The thermal analyses were recorded using the Pyris software, Version 11.1.1.0492 [13].

#### 2.5.3. X-ray Powder Diffraction (XRD)

XRD samples were evaluated by an Ultima IV diffractometer (Rigaku Corporation, Tokyo, Japan) over 3–30° 2θ range at 0.5 deg./min scan speed. The tube anode was Cu with Ka = 0.154 nm monochromatized with a graphite crystal. The pattern was collected at tube voltage (40 kV) and tube current (40 mA) in step scan mode (step size 0.02°, counting time 1 s per step) [18].

#### 2.5.4. Droplet Size Analysis and Zeta Potential of Liquid and Solid SNEDDS

Drug-loaded liquid/solid SNEDDS and combination of (SD + drug-free SNEDDS) samples were dispersed in distilled water at ratio 1:1000 *w*/*w*, stirred for 5 min at 1000 rpm to ensure uniform formulation dispersion [18]. Prior to examination, the aqueous dispersion was centrifuged at 10,000 rpm to separate the supernatant from undissolved particles which arise from the solid excipients and might interfere with the droplet size of the aqueous dispersion. Finally, the mean particle size and polydispersity index (PDI) of each formulation were measured by photon correlation spectroscopy (PCS) using a Zetasizer Nano ZS (Malvern Instruments, Malvern, Worcestershire, UK). The particle size of the aqueous dispersions was evaluated using dynamic light scattering (DLS) mode at the 25 °C. Zeta potential of each formulation was evaluated by laser doppler velocimetry (LDV) mode using the same Nano ZS at 25 °C. The average particle size, polydispersity index and zeta potential were determined by taking the mean of six replicates [30].

#### 2.5.5. Transmission Electron Microscopy

Diluted solid SNEDDS formulation was sonicated for 10 min prior to grid preparation. One copper grid (Ted Pella) of 300 mesh with support film of carbon type-B was kept on a clean parafilm. Then, one drop of sonicated formulation was poured upon the grid and allowed to settle the particles for at least 10 min. Later, the grid was removed from the parafilm and left to dry overnight with a proper covering to avoid any contamination from the atmosphere. The completely dried grid was mounted in the sample holder and viewed under JEOL JEM1010 transmission electron microscope (Tokyo, Japan) at an operating voltage of 80 kV.

#### 2.5.6. In Vitro Dissolution Tests

CN formulations were tested at pH 6.8 dissolution medium to mimic the release in the worst expected conditions that equivalent to the elevated gastric pH environment [3,4].

The dissolution tests were performed using an automated USP Type II dissolution apparatus (UDT-814, LOGAN Inst. Corp., Franklin, NJ, USA) at 50 rpm paddle speed. The dissolution medium was composed of 500 mL of phosphate buffer at pH 6.8. The buffer was prepared according to EUROPEAN PHARMACOPOEIA 7.0 and composed of 0.1% *w*/*v* potassium dihydrogen phosphate, 0.2% *w*/*v* dipotassium hydrogen phosphate and 0.85% *w*/*v* sodium chloride. The dissolution medium was maintained at 37 °C and involved no enzymes. The formulation for the equivalent amount of drug (25 mg CN) was weighed and the experiment was conducted using three replicates; 2 mL serial samples were withdrawn at 5, 10, 15, 20,30, 60 and 120 min. After centrifugation, an aliquot of the supernatant was diluted in acetonitrile and assayed by the UPLC [26]. Formulation performance was compared based on the dissolution efficiency (DE)% [31].

#### 2.5.7. CN Quantification by UPLC Assay

CN was quantified by using a validated UPLC reversed-phase method [26], with minor modifications. The mobile phase composition was changed to 0.5% trifluoracetic acid: acetonitrile (60:40) and the run time increased to 5 min, to allow for higher resolution between the intact drug and degradation product peaks. Peak separation was achieved using an Acquity^®^ UPLC BEH C18 (2.1 × 50 mm, 1.7 µm) column connected with an Acquity guard filter and the flow rate maintained at 0.25 mL/min. The UV detector was set at 251 nm.

### 2.6. Statistical Analysis

SPSS 26 software was utilized to test the significance of the data. The influence of drug-loading and solidification on were examined by Paired *t*-test. Droplet size, zeta potential and dissolution efficiency were compared using one-way ANOVA followed by Post Hoc tests “LSD”. A value of *p* < 0.05 was denoted as significant throughout the study [17].

## 3. Results

### 3.1. Preparation and Optimization of Solid SNEDDS and SDs

Both drug-free and drug-loaded solid SNEDDS prepared by adsorption onto Neusilin^®^ US2 were completely solidified as free flowing powder with no signs of particle agglomeration. As expected, the absence of CN from the solid SNEDDS did not have any adverse effect on the solidified powder flow outcomes. Regarding CN-SD, the drug was successfully encapsulated within both FD-SD and MW-SD with encapsulation efficiencies of 86% ± 17 and 83% ± 14, respectively.

### 3.2. Characterization of Solid SNEDDS and SDs

#### 3.2.1. Powder Properties

Pure Neusilin^®^ US2 (NUS) showed excellent flow properties among different powder flow characterization factors (Table 2). Upon SNEDDS adsorption on NUS, the resulting S-SNEDDS were completely solidified as free flowing powder and showed acceptable flow properties that ranged from fair to good, according to USP guidelines chapter [32].

#### 3.2.2. Differential Scanning Calorimetry (DSC)

**Figure 2:**
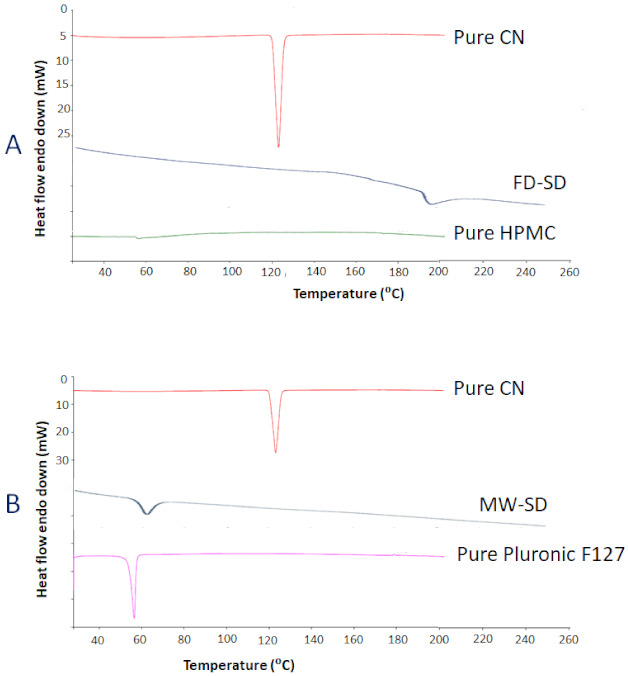
DSC chromatograms (**A**) FD-SD and (**B**) MW-SD of CN formulations. FD-SD: freeze drying induced solid dispersion, MW-SD: microwave induced solid dispersion, CN: cinnarizine.

The pure CN demonstrated a sharp endothermic peak at 125 °C (Figure 2) which confirmed the crystalline state of CN [33]. Both pure HPMC and FD-SD showed complete disappearance of CN peak at its expected temperature range. FD-SD showed a small broad peak at 200 °C. On the other hand, both pure Pluronic F127 (PL-127) and MW-SD showed new endothermic peak at 50–60 °C, while the CN peak was completely disappeared.

#### 3.2.3. X-ray Diffraction (XRD)

**Figure 3:**
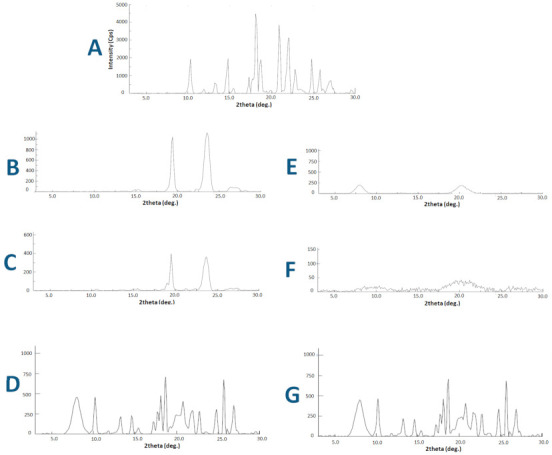
XRD analysis of (**A**) pure CN, (**B**) Pure PL-127, (**C**) MW-SD, (**D**) CN/ PL-127 physical mixture, (**E**) pure HPMC, (**F**) FD-SD formulations and (**G**) CN/HPMC physical mixture. FD-SD: freeze drying induced solid dispersion, MW-SD: microwave induced solid dispersion.

Similar to DSC, the pure CN exhibited typical X-ray diffraction peaks at 3° to 30° (2θ) (Figure 3) confirming its crystalline state. Both pure HPMC and FD-SD showed complete absence of sharp CN diffraction peaks. Alternatively, pure PL-127 and MW-SD showed two sharp peaks at 18–25° (2θ) while CN diffraction peaks were completely disappeared. Both CN/PL-127 and CN/HPMC physical mixtures showed CN typical X-ray diffraction peaks at 3° to 30° (2θ).

#### 3.2.4. Droplet Size Analysis and Zeta Potential of Liquid/Solid SNEDDS

**Figure 4:**
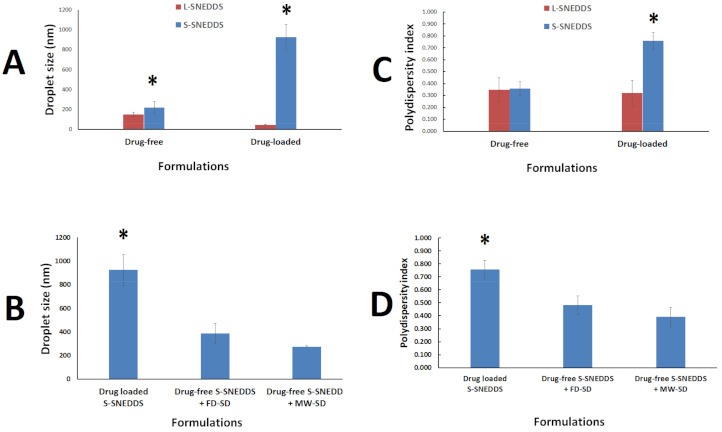
Droplet size and polydispersity index (PDI) of diluted SNEDDS formulations. (**A**,**B**) show the influence of drug-loading and combined solid SNEDDS on droplet size, respectively. (**C**,**D**) show the influence of drug-loading and combined solid SNEDDS on polydispersity index, respectively. * denotes significant (*p* < 0.05) difference from counterpart formulations. Data are expressed as mean ± SD, *n* = 6.

Drug-free SNEDDS showed significant increment of droplet size upon formulation solidification while drug-loaded SNEDDS showed significant increment of both droplet size and PDI upon formulation solidification (Figure 4A,C). Interestingly, the combination of drug-free solid SNEDDS/FD-SD and drug-free solid SNEDDS/MW-SD showed significant reduction of both formulation droplet size and PDI, compared to the conventional drug-loaded solid SNEDDS prepared in the current studies (Figure 4B,D).

**Figure 5:**
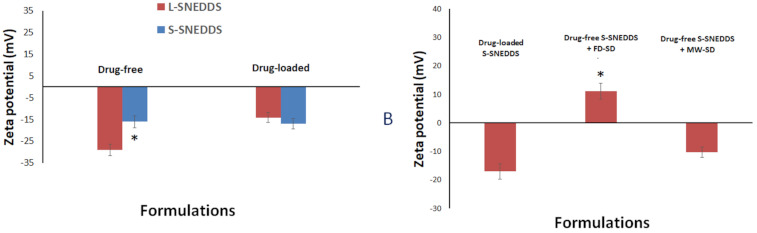
Influence of (**A**) drug-loading and (**B**) combined solid SNEDDS on formulation zeta potential. * denotes significant (*p* < 0.05) difference from counterpart formulations. Data are expressed as mean ± SD, *n* = 6.

On the other hand, only drug-free SNEDDS showed significant decrease of zeta potential absolute value while drug-loaded SNEDDS showed no significant change upon solidification (Figure 5). Interestingly, the combination of drug-free solid SNEDDS + FD-SD shifted the zeta potential value to the positive region and showed significant (*p* < 0.05) difference from drug-loaded solid SNEDDS, as well as drug-free solid SNEDDS + MW-SD.

#### 3.2.5. Transmission Electron Microscopy

**Figure 6:**
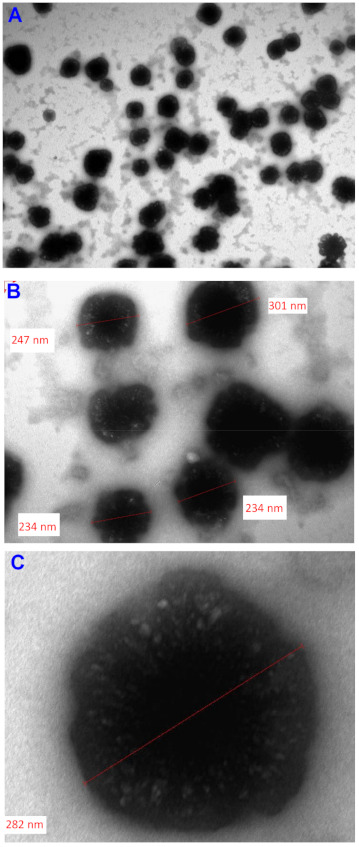
TEM images of (FD-SD + drug-free S-SNEDDS) at (**A**) 30,000, (**B**) 100,000 and (**C**) 200,000× magnifications.

Transmission electron microscopy images of diluted representative SNEDDS formulation (FD-SD + drug-free S-SNEDDS) is shown in Figure 6. The droplet size was found to be 200–300 nm, which is in proximity with the results obtained by the Zetasizer Nano particle sizing systems. The nanoemulsion droplets were discrete, spherical in shape with distinctive outer layer that represents the surfactant molecules agglomeration around the oil nanodroplets (Figure 6C).

#### 3.2.6. In Vitro Dissolution

##### Effect of Neusilin^®^ US2 on Drug Release from SNEDDS

**Figure 7:**
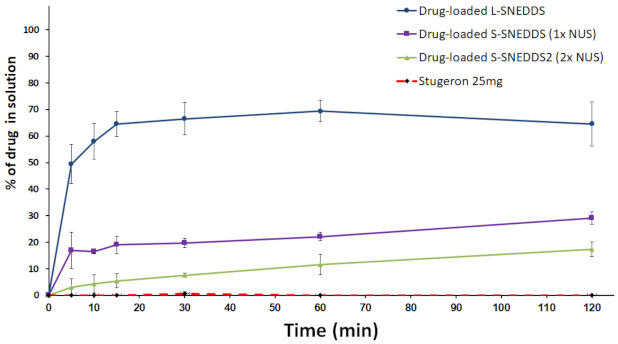
Effect of Neusilin^®^ US2 on CN release from SNEDDS at pH 6.8. Data are expressed as mean ± SD. *n*= 3 for drug-loaded L-SNEDDS and Stugeron while *n* = 6 for drug-loaded S-SNEDDS (1× NUS) and drug-loaded S-SNEDDS2 (2× NUS).

At pH 6.8, Stugeron tablet showed negligible dissolution (<1%) up to 2 h (Figure 7). In contrast, drug-loaded L-SNEDDS showed enhanced dissolution efficiency (65% release) at 2 h. SNEDDS solidification by NUS led to significant (*p* < 0.05) decline in CN dissolution efficiency. Drug-loaded S-SNEDDS (1× NUS) and (2× NUS) showed up to 29 and 17% CN release, respectively.

##### Effect of SD + L-SNEDDS on Drug Release

**Figure 8:**
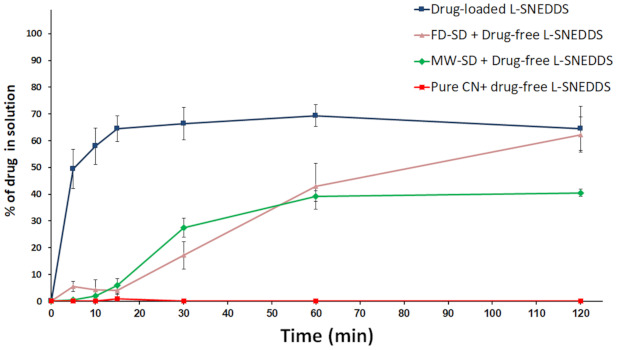
Effect of (SD + L-SNEDDS) combination on CN dissolution at pH 6.8. Data are expressed as mean ± SD. *n* = 3 for drug-loaded L-SNEDDS; *n* = 4 for MW-SD + Drug-free L-SNEDDS; *n* = 6 for FD-SD + Drug-free L-SNEDDS and Pure CN + drug-free L-SNEDDS.

The combination of (Pure CN + drug-free L-SNEDDS) showed negligible dissolution at pH 6.8 (Figure 8). On the other hand, the combination of (drug-free L-SNEDDS + MW-SD) and (drug-free L-SNEDDS + FD-SD) showed significant (*p* < 0.05) enhancement of CN dissolution up to 40% and 62%, respectively. In contrast, the drug loaded L-SNEDDS showed the maximum CN release of 65%.

##### Effect of SD + S-SNEDDS on Drug Release

**Figure 9:**
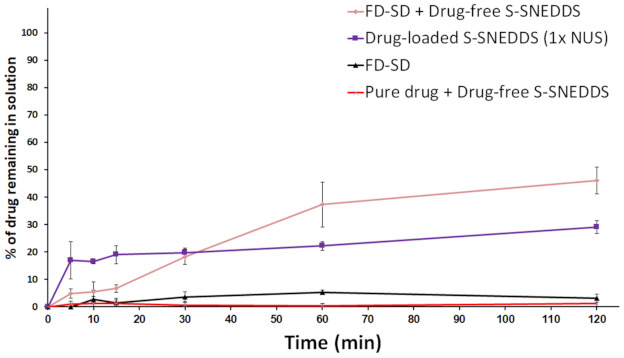
Effect of (SD + S-SNEDDS) combination on CN dissolution at pH 6.8. Data are expressed as mean ± SD, *n* = 6.

FD-SD showed ~5% CN dissolution up to 2 h at pH 6.8 (Figure 9). On the other hand, (FD-SD + drug-free S-SNEDDS) showed up to 46% dissolution compared to drug-loaded S-SNEDDS that showed a maximum of 29% CN release, up to 2 h. As expected, (pure CN + drug-free S-SNEDDS) showed the negligible dissolution at pH 6.8.

## 4. Discussion

At normal gastric conditions, WB-PWSD experiences its first exposure at the lower pH gastric environments which favor good CN solubilization. Subsequently, upon shifting to the intestinal environments, WB-PWSD tends to precipitate due to unfavorable higher pH conditions. However, this may not exactly occur in vivo due to sink conditions and fast absorption capacity available in the intestinal tract.

At hypochlorhydria, WB-PWSD would unexpectedly face the unfavorable high pH environment from its initial approach to the gastro-intestinal tract. Therefore, this drug would be deprived from its favorable low pH environments leading to extremely poor drug solubility and dissolution at higher pH conditions. Such drug properties would lead to exponential decline in drug solubility, dissolution and bioavailability upon gastric pH increase.

The conventional design of drug-loaded SNEDDS involves dissolving the drug within the SNEDDS components during formulation preparation. Upon aqueous dispersion, the drug-loaded SNEDDS would spontaneously self-emulsify and try to keep the drug in solution. However, this SNEDDS drug loading technique could be challenging if it fails to keep the drug in solubilized state. In the current study, the combined (SD + drug-free SNEDDS) were designed based on a novel approach where the PWSD was completely isolated from the solid SNEDDS. This approach was based on hypothesizing that, upon aqueous dispersion of the system, drug-free SNEDDS would self-emulsify to produce enormous nano-sized micelles. Simultaneously, the amorphized drug would tend to migrate into the formed nanoemulsion droplets while the crystalline drug would fail to do the same. This point is especially critical in case of WB-PWSD at higher pH media [18].

To proof this hypothesis, two solid dispersion techniques were examined within the current study, namely, microwave irradiation (MW-SD) and freeze drying (FD-SD) solid dispersion. From the characterization results, the DSC and XRD data showed successful CN amorphization within MW-SD and FD-SD as confirmed by the disappearance of CN characteristic peaks from the chromatograms (Figure 2 and Figure 3). In the DSC data, the endothermic peak at 53–56 presented with pure PL-127 and MW-SD corresponds to the characteristic peak of PL-127 as reported earlier (Figure 2B) [34]. Similarly, the characteristic XRD peaks at 18–25° (2θ) corresponds to PL-127 (Figure 3B,C) [34] while CN diffraction peaks were completely disappeared. In addition, the presence of CN diffraction peaks in both CN/PL-127 and CN/HPMC physical mixtures confirmed that drug amorphization occurred only in CN-SD samples. The DSC and XRD findings are in good correlation and confirm successful CN amorphization within the two prepared solid dispersions FD-MW and MW-SD.

The observed droplet size increase upon solidification could be due to the presence of the insoluble silica carrier (NUS) which might have interfered with the formed nanoemulsion droplets and led to significant droplet size growth. On the other hand, combination of SD with drug-free solid SNEDDS showed potential role to counter act the negative influence of solidification on formulation droplet size. In fact, both the FD-SD and MW-SD combination showed 50% and 70% reduction in the droplet size of solidified SNEDDS.

Zeta potential is another parameter in evaluating emulsification efficiency. The significance of zeta potential value could be linked with nanoemulsion stability. Colloids with high zeta potential (negative or positive) are electrically stabilized and vice versa. Most of the SNEDDS formulations exhibit negative zeta potential values which might be owing to the presence of non-ionic surfactants, adsorption of anionic species to the droplet surfaces, or the existence of some anionic impurities in the surfactant (such as free fatty acids) [21]. Interestingly, the combination of (drug-free SNEDDS + FD-SD) led to significant shift of zeta potential value to positive value which might be owing to the presence of HPMC within the formulation. This finding is interesting and could have great potential in enhancing drug absorption from the combined (SNEDDS + SD) system [35].

Drug-loaded L-SNEDDS represent a powerful tool to produce a significant and pH-independent enhancement of PWSD dissolution. However, previous studies revealed significant CN degradation within L-SNEDDS formulations upon storage as well as formulation discoloration due to rancidity [15,16]. Therefore, the current study focused on achieving a novel solidification technique to solve the reported limitation of L-SNEDDS along with maintaining enhanced drug dissolution particularly at the most challenging conditions of elevated pH levels. In the current study, drug-loaded S-SNEDDS showed significant inhibition of CN release compared to L-SNEDDS. SNEDDS solidification by NUS led to significant (*p* < 0.05) decline in CN dissolution. The latter was inversely proportional to NUS amount in the formulation (Figure 7). In fact, drug-loaded S-SNEDDS might experience slower rate of CN degradation but it is not expected to completely stop degradation because the drug would still be exposed to SNEDDS excipients even at solid stage [18]. On the other hand, the isolation between drug-free SNEDDS and CN has great potential to enhance drug stability within the formulation. Accordingly, this study was further justified to evaluate the feasibility of this technique.

An in vitro dissolution study was designed to examine the combination of two capsules [capsule #1 containing pure CN + capsule #2 containing drug-free liquid SNEDDS] at pH 6.8 (Figure 8). The system showed negligible CN dissolution due to inability of pure crystalline CN to partition into drug-free nanoemulsion droplets upon aqueous dispersion (Figure 8). In contrast, the combination of (SDs + drug-free L-SNEDDS) showed significant enhancement of CN dissolution due to successful partitioning of CN into micelles which can be strongly correlated with successful drug amorphization as confirmed by DSC and XRD results (Figure 2 and Figure 3). The CN solid dispersion provided transient protection of the drug from the unfavorable aqueous environment along with facilitating its migration towards favorable nanoemulsion droplets generated by drug-free SNEDDS (Figure 8). Although self-nanoemulsification process was quite faster, drug migration towards the nanoemulsion droplets occurred at slower rates as represented by slower drug release in case of combined systems compared to drug-loaded SNEDDS (Figure 8). However, by the end of the dissolution experiment, (FD-SD + drug-free L-SNEDDS) showed similar drug dissolution to drug-loaded L-SNEDDS.

FD-SD were examined alone to evaluate its sole role in CN dissolution enhancement. The results showed that FD-SD presented only 4% dissolution efficiency (DE) which reflects the insufficiency of freeze-dried SD alone to enhance CN dissolution (Figure 9). In contrast, (FD-SD + drug-free S-SNEDDS) showed 880% and 160% release enhancement compared to uncombined FD-SD and drug-loaded S-SNEDDS, respectively. In addition, (FD-SD + drug-free S-SNEDDS) provides ultimate drug isolation from the adsorbent and/or SNEDDS excipients, which could avoid the unfavorable development of physical bonds between the drug and the adsorbent that might get strengthened upon storage. These data prove the potential effect of SD + drug-free S-SNEDDS combination in providing good CN dissolution enhancement along with robust stability profile on SNEDDS solidification as well as complete drug isolation from SNEDDS excipients.

Based on these findings, the claimed hypothesis was proven and the system can be developed so that the drug-free S-SNEDDS would be totally separated from CN loaded SD to minimize drug interaction with the formulation excipients and/or the adsorbent. The developed formulation could be filled using capsule-in-capsule strategy where CN-SD and drug-free S-SNEDDS would be filled in two different capsules [36]. Alternatively, drug-free S-SNEDDS might be compressed into tablets followed by layering of CN-SD as a separate coating layer on top of the SNEDDS tablet [13].

## 5. Conclusions

Solid self-nanoemulsifying drug delivery systems (S-SNEDDS), prepared by adsorption, offer good option to enhance drug dissolution and stability. However, the adsorbent such as Neusilin^®^ used in this technique significantly retards drug release from SNEDDS formulation. In the current study, the self-nanoemulsifying formulations were successfully solidified alone and combined with pure weakly basic drug cinnarizine. Combined (Drug-free S-SNEDDS + SD) offers a novel approach to overcome the negative impact of adsorbent on drug release from SNEDDS. In addition, the developed dosage form design could be the potential technique to enhance the absorption of unstable weakly basic drugs within the patients suffering from hypochlorhydria.

## Figures and Tables

**Table 1:** Development of various liquid and solid formulations for CN using lipid-based excipients and inorganic silica materials.

Excipients	Formulations *						
	Drug-Free L-SNEDDS	Drug-Loaded L-SNEDDS	Drug-Free S-SNEDDS	Drug-Loaded S-SNEDDS (1× NUS)	Drug-Loaded S-SNEDDS (2× NUS)	MW-SD	FD-SD
CN	-	8	-	4	2.7	10	20
Oleic acid	25	23	12.5	11.5	7.7	-	-
Imwitor I308	25	23	12.5	11.5	7.7	-	-
Cremophor El	50	46	25	23	15.3	-	-
Neusilin US2	-	-	50	50	66.7	-	-
HPMC E3	-	-	-	-	-	-	80
Pluronic F127	-	-	-	-	-	90	-
SUM	100	100	100	100	100	100	100

* All the excipients’ quantities are expressed as *w*/*w* %. Abbreviations: L-SNEDDS and S-SNEDDS: liquid and solid self-nanoemulsifying drug delivery systems, respectively. MW-SD: microwave induced solid dispersion. FD-SD: freeze dried solid dispersion. Drug-loaded and drug-free liquid SNEDDS were solidified using Neusilin^®^ US2 at 1:1 (1× NUS) and 1:2 (2× NUS) ratios.

**Table 2:** Evaluation of powder flow properties of drug-free S-SNEDDS as compared to Neusilin US2 powder.

Test	Pure NUS		Drug-Free S-SNEDDS	
	Result	Flow Property *	Result	Flow Property *
Angle of repose	24.7 ± 0.3	Excellent	33.6 ± 0.1	Good
Bulk density	0.19 ± 0.0		0.29 ± 0.0	
Tapped density	0.20 ± 0.0		0.35 ± 0.0	
Compressibility index	6.49 ± 0.2	Excellent	17.07 ± 0.37	Fair
Hausner ratio	1.07 ± 0.0	Excellent	1.21 ± 0.0	Fair

* Categorized according to USP 35, Powder flow chapter [32]. Data are expressed as mean ± SD, *n* = 3.

## Data Availability

Not available.
